# Experimental Setup and Measuring System to Study Solitary Wave Interaction with Rigid Emergent Vegetation †

**DOI:** 10.3390/s19081787

**Published:** 2019-04-14

**Authors:** Davide Tognin, Paolo Peruzzo, Francesca De Serio, Mouldi Ben Meftah, Luca Carniello, Andrea Defina, Michele Mossa

**Affiliations:** 1 Department of Civil, Environmental and Architectural Engineering, University of Padova, via Loredan, 20, 35131 Padova, Italy; paolo.peruzzo@unipd.it (P.P.); luca.carniello@unipd.it (L.C.); andrea.defina@unipd.it (A.D.); 2 Department of Civil, Environmental, Land, Building Engineering and Chemistry, Polytechnic University of Bari, via Orabona, 4, 70125 Bari, Italy; francesca.deserio@poliba.it (F.D.S.); mouldi.benmeftah@poliba.it (M.B.M.); michele.mossa@poliba.it (M.M.); 3 CoNISMa, National Interuniversity Consortium of Marine Sciences, Piazzale Flaminio 9, 00196 Roma, Italy

**Keywords:** laboratory experiments, solitary wave, wave-vegetation interaction, advanced hydrometry

## Abstract

The aim of this study is to present a peculiar experimental setup, designed to investigate the interaction between solitary waves and rigid emergent vegetation. Flow rate changes due to the opening and closing of a software-controlled electro-valve generate a solitary wave. The complexity of the problem required the combined use of different measurement systems of water level and velocity. Preliminary results of the experimental investigation, which allow us to point out the effect of the vegetation on the propagation of a solitary wave and the effectiveness of the measuring system, are also presented. In particular, water level and velocity field changes due to the interaction of the wave with rigid vegetation are investigated in detail.

## 1. Introduction

It is widely recognized that vegetation plays a pivotal role in the preservation and restoration of coastal environments, since it controls the sedimentation and transport, as well as it contributes to dissipating wave energy [1,2,3,4,5,6,7,8,9,10,11,12]. Concerning the latter aspect, as a consequence of the catastrophic tsunami event on the coast of South-East Asia in 2004, many studies have focused on the protective action of the coastline provided by mangrove forests [13,14,15,16,17,18].

Mangroves, in fact, can effectively protect the coastline from the action of wind and tidal waves [19]; however, dedicated studies suggest that tsunamis and storm surges behave differently. For example, as water height of severe tsunami and surges increases, the attenuation provided by mangrove forests is likely to reduce. The long period of tsunami waves may also influence the mitigation provided by mangroves because plants could be already damaged or uprooted as the wave continues to propagate through the coastal forest [20,21].

To better understand the action exerted by mangrove forests, the quantification of the tsunami wave attenuation, as well as the drag arising within mangroves have been investigated both numerically and experimentally, but, regardless of the approach employed, the efficacy of the mangroves is still an open question [22,23,24,25,26,27]. For instance, Kathiresan and Rajendran [28] demonstrated the ability of mangrove vegetation to protect the coastline from tsunamis’ fury; meanwhile, Kerr et al. [29] stated that this sense of protection from large tsunamis appears to be unrealistic.

These dissimilarities in the outcomes can be explained by the complexity of the phenomena that characterize the interaction between waves and mangroves, which is often simulated through a series of small amplitude waves propagating within a canopy of artificial vegetation, in most of the cases mimicked by rigid dowels [23,24,25,30,31,32,33,34]. Such modeling of the problem is quite far from reality since the tsunami approaching the coast can be seen as a solitary wave, rather than a small wave. The latter only propagates its form; meanwhile, the orbital paths of water particles in a solitary wave are open; thus, water mass transport occurs. The reproduction of different types of waves in shallow water in physical models is a quite demanding task and should be carefully addressed [35,36].

The use of laboratory-scale models, despite the considerable simplification necessary for the experiments being feasible, is a consistent approach to isolate the effects of different wave and plant characteristics, thanks to the controlled conditions, as well as to validate numerical models (i.e., [37,38]).

The present work aims to describe a properly-designed experimental setup to reproduce and study the propagation of a solitary wave in the presence of emerging rigid cylinders mimicking the propagation of tsunamis through mangrove forests. The complex process of drag and flow separation is analyzed by means of several types of equipment able to measure different quantities; namely, ultrasonic probes for the water level, Acoustic Doppler Velocimeters (ADVs) for local velocity, and a Particle Image Velocimetry (PIV) system for the velocity field in a frame. Many previous experiments have been carried out by using ADVs or PIV separately. The advantages of using ADVs are based on their high frequency rate (100 ÷ 200 Hz), providing also information about turbulent aspects of the phenomenon. However, it is an intrusive instrument presenting limitations especially close to obstacles and channel bottom/walls. PIV overcomes this limitation, but it is often insufficient for a detailed turbulence analysis because common PIV systems generally have relatively low acquisition frequency (in the range of 5 ÷ 50 Hz). Therefore, the coupling of ADVs and PIV has an intrinsic value as it allows us accurate measurements near boundaries and obstructions with high-frequency sampling rates. To our knowledge, this is the first example of integrated systems that measure the dynamics of a solitary wave in this context.

## 2. Experimental Setup

**Figure 1:**
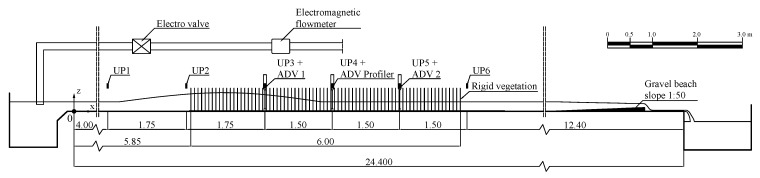
Side view of the experimental setup, with the positions of the Ultrasonic Probes (UPs) and the Acoustic Doppler Velocimeters (ADVs).

Experiments are carried out in a laboratory rectangular flume at the Department of Civil, Environmental, Land, Building Engineering and Chemistry (DICATECh) of the Polytechnic University of Bari (Italy) (Figure 1). The channel is 25 m long, 0.40 m wide, and 0.50 m high, and it is made of Plexiglas to guarantee optical access. Water recirculates through the channel in two partially separated circuits, each one fed by a different tank. More precisely, the main circuit maintains steady flow conditions via a first constant head tank, whilst a secondary tank can discharge up to 80 L/s by regulating a software-controlled electro-valve, and thus generating a wave due to the flow rate change. A triangular sharp-crested weir at the downstream tank is used to estimate the steady flow rate, whereas a more accurate measure of the flow rate from the secondary tank is provided by an electromagnetic flow meter, placed upstream of the channel. The water level at the end of the flume is controlled by a sloping (1:50) gravel beach. For further details, see [39].

A six meter-long vegetation canopy is housed within the flume at 5.85 m from the channel inlet. Vegetation consists of a set of rigid steel cylinders with diameter d=3 mm, inserted into six previously drilled Plexiglas panels. Different cylinder densities and patterns are obtained using the regular grid of holes longitudinally and transversally spaced with the same axis-to-axis distance (i.e., s=4 cm).

The free surface variation in the flume is measured by six ultrasonic probes at a 100 Hz sampling rate. The probes are located at x= 4.00 m, 5.75 m, 7.50 m, 9.00 m, 10.50 m, and 12.00 m, so that the first two probes measure the approaching wave height, and the other four sensors measure the wave attenuation along the canopy (see Figure 1, which includes the *x*, *z* reference coordinate system).

Two different systems are used to measure the velocity within the canopy. The single point velocity is measured by means of two 3D Acoustic Doppler Velocimeters (ADVs), whose sampling rate, velocity range, and sampling volume are set equal to 100 Hz, ±1.00 m/s, and 7 mm, respectively, in order to achieve a velocity accuracy of ±1%. A further three-dimensional ADV Profiler, which measures velocity components in 33 cells with 1 mm of vertical extent at a 100 Hz sampling rate, is used to reconstruct nearly instantaneous vertical velocity profiles. During the ADVs’ measurements, a correlation of the signal around 90% and a signal-to-noise ratio around 12 dB are achieved, thus proving the good quality of the signal itself. Figure 1 shows that the three ADV measurement systems are located in the canopy at the same position of the ultrasonic probes, i.e., x= 7.50 m, 9.00 m, 10.50 m, in order to obtain water level and velocity measures simultaneously.

The flow velocity field within the canopy is also measured by 2D Particle Image Velocimetry (PIV). The system consists of a timer box and a timer card cable box, triggering a FlowSense EO 4M-32 camera (frame rate of 32 Hz) with a dual power Laser Continuum Minilite (energy of 25 mJ at 15 Hz). The PIV, with the camera mounted to record from the bottom view, is setup to investigate turbulence structures in the horizontal plane at x= 9.00 m. The PIV system is handled in double-frame mode, and the total number of analyzed pair of frames is 381. The PIV sampling frequency is settled to its maximum (i.e., 16 Hz), while the time interval between two images of the same pair is 500 μs. Thus, the total acquisition time is 24 s for each measurement in the target area of the channel. The obtained images have dimensions of 2072 × 2072 pixels, corresponding to 132 mm × 132 mm, after the calibration. The interrogation area is 16 × 16 pixels, thus, the velocity vectors are computed on points spaced 1 mm, providing a very high spatial resolution in the measurement.

For simulating the tsunami propagation, we generate a solitary wave, reaching a flow peak of 85 L/s, as the sum of a steady flow of 10 L/s and an unsteady flow produced by linearly opening and closing the electro-valve for 10 s and 20 s, respectively.

**Figure 2:**
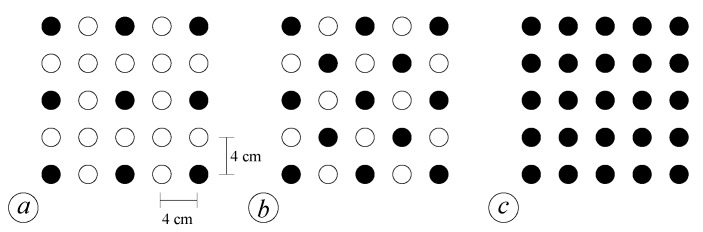
Vegetation patterns for the three tested configurations: (**a**) plant density n=156.25 cylinder/m^2^; (**b**) plant density n=312.5 cylinder/m^2^; (**c**) plant density n=625 cylinder/m^2^.

Four scenarios are investigated. In the first scenario, the wave propagates in the absence of vegetation; the other three scenarios consider different vegetation densities *n* (i.e., n= 156.25, 312.5, and 625 cylinder/m^2^) with different vegetation patterns (see Figure 2). For each scenario, we perform at least ten series of experiments, and each series of experiments counts 30 runs in order to analyze the data according to a phase-resolved approach.

**Figure 3:**
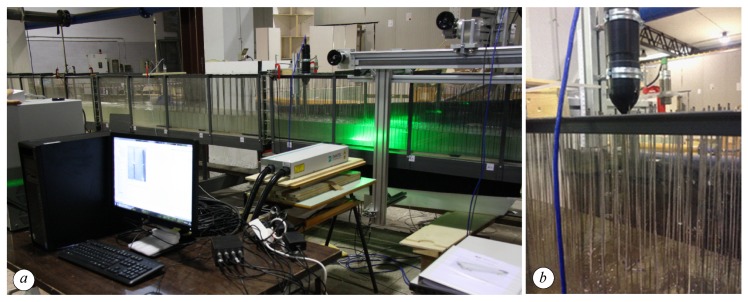
(**a**) View of the flume section with the Particle Image Velocimetry (PIV); (**b**) the particulars of an ultrasonic probe and ADV.

An overview of the experimental set-up is shown in Figure 3.

## 3. Results and Discussion

**Figure 4:**
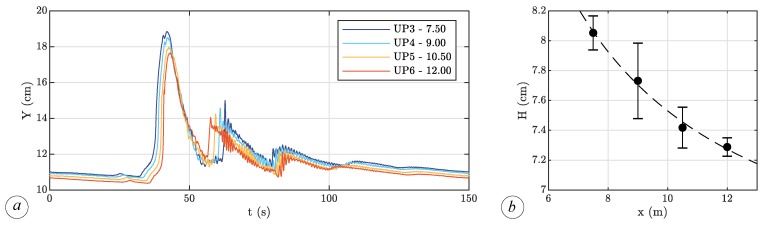
(**a**) Water level *Y* measured in 4 sections, without vegetation; (**b**) reduction of the wave height *H* without vegetation.

The first scenario is aimed at understanding the characteristics of the wave in the absence of vegetation. Examples of water level during the propagation of the solitary wave within the flume and without vegetation are shown in Figure 4, where the time-varying water level is measured by the four ultrasonic probes, located where the cylinder array will be housed in the scenarios reproducing the presence of vegetation. Each probe measures the typical symmetric, bell-shaped form of the water level at the passage of the solitary wave. As provided by the solitary wave theory, the propagation of the wave occurs entirely above the undisturbed water surface. The data collected clearly show the presence of a first reflected wave due to the sloping beach at the end of the flume (60 s < *t* < 80 s) and a second one reflected by the upstream tank (80 s < *t* < 95 s). However, these reflected waves do not affect the main incoming wave, since they both reach the area of interest after the main incoming wave has almost completely passed. Removing the sloping beach could reduce the reflection phenomena, but would make it impossible to control the downstream boundary condition.

The spatial variation of the wave height in bare soil conditions (i.e., without vegetation) is shown in Figure 4b. The water level decreasing is more than linear along the considered 4.5 m long reach, achieving a maximum reduction of about 12%. In the presence of vegetation, the free surface varies similarly to the bare soil case, and for sake of brevity, this result is not reported here.

**Figure 5:**
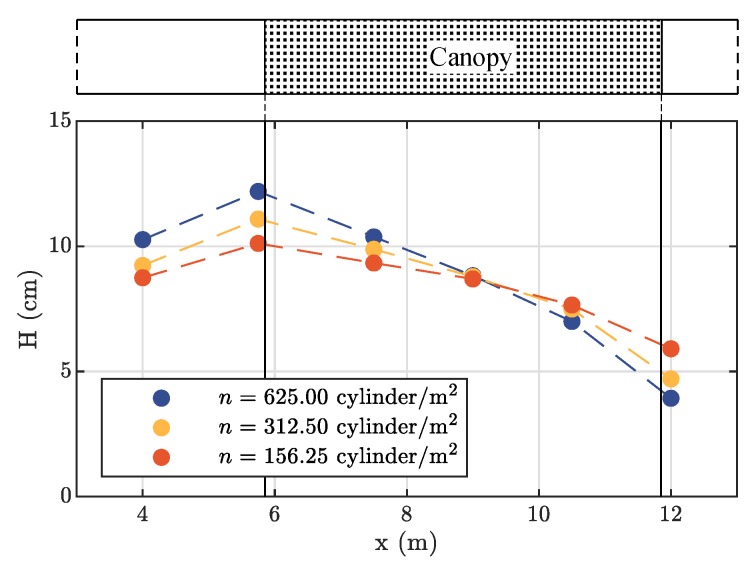
Reduction of the wave height *H* with different vegetation densities.

Experimental results referring to the vegetated scenarios show that the canopy density strongly affects the spatial reduction of the wave peak. Figure 5 shows the wave attenuation along the vegetated reach for the three investigated vegetation densities. In accordance with the literature [8,40,41,42], a stronger attenuation of the wave occurs with the increasing of the density *n*. In fact, the wave height at the end of the vegetated area compared to the incoming wave height reduces by about 40% for n=156.25 cylinder/m^2^ and up to more than 60% for n=625 cylinder/m^2^. The observed reduction of the solitary wave height is quite far from the hyperbolic decrease provided by the linear wave theory [41], confirming the idea that a reliable representation of the impact of mangroves on tsunamis cannot leave a careful wave generation out of consideration. Moreover, the wave height upstream of the canopy increases before impacting the vegetation. This supports the idea that vegetation could behave as a porous barrier for the incident solitary wave. These aspects deserve further theoretical and experimental analysis, in which the combined effect of water level and flow velocity needs to be taken into account to determine the wave attenuation.

**Figure 6:**
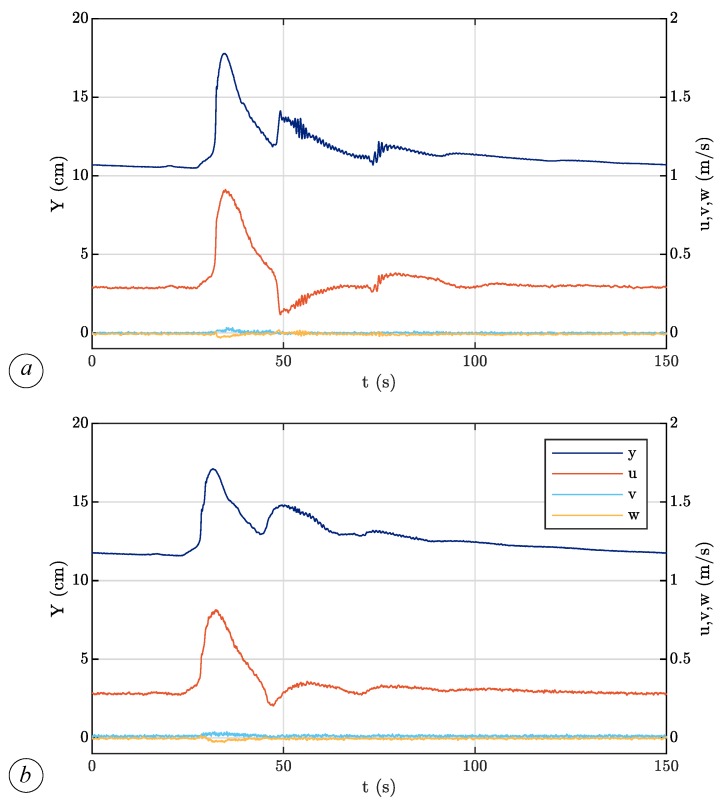
Water level (*Y*) and velocity components (*u*, *v*, *w*) measured at x= 10.5 m and z= 2 cm (**a**) without vegetation and (**b**) in the presence of vegetation with density n=156.25 cylinder/m^2^.

The ADV acquisitions allow us to couple water level and velocity data. In Figure 6, we can compare water level and the three velocity components at x= 10.5 m and z= 2 cm as a function of time for the bare soil (Figure 6a) and in the presence of vegetation with density n=156.25 cylinder/m^2^ (Figure 6b). In both cases, the peak of longitudinal velocity *u* slightly precedes the water level peak. The transverse velocity component, *v*, and the vertical one, *w*, are negligible compared to the longitudinal velocity. By comparing the two cases, we observe that the vegetation smoothing effect is more evident for water levels than for flow velocities.

**Figure 7:**
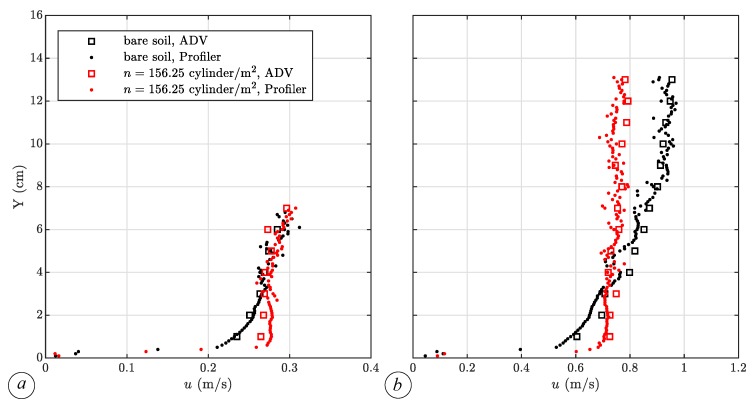
Vertical profile of the longitudinal velocity component *u* at x= 9.00 m on the channel axis, in bare soil conditions (black markers) and with vegetation (red markers), during base flow conditions (**a**) and at the wave peak (**b**). Small dots denote the ADV Profiler output, and squares denote ADV measurements.

Measures at wave crest conditions point out the advantages of simultaneous water level and velocity measurements. Indeed, ADV outputs are meaningful only if the sensor’s measurement volume is completely submerged, and this condition can be easily detected comparing ultrasonic probe measurements. In this way, we can recognize the true signal from the noise recorded when the ADV sensors are out of the water, and thus, we can investigate the velocity field even in points above the base flow water level. Figure 7 shows a comparison between the vertical profiles of the longitudinal velocity *u* at x= 9.00 m on the channel axis, in bare soil conditions and with vegetation density n=156.25 cylinder/m^2^, both during base flow conditions and at the wave peak. It is worthwhile noting that the velocity measurements in the second case are also reported in the wave crest, namely between 7 cm and 14 cm (see Figure 7b), discerned by means of the analysis of the signal provided by the water level probe. Measurements with the ADV Profiler overlap those of the single point ADV, but they have a finer spatial resolution. In bare soil conditions, the profile clearly follows a logarithmic law, with a maximum value of 0.3 m/s during base flow conditions and 0.9 m/s at the wave peak. Meanwhile, with vegetation, the velocity is nearly uniform over depth (equal to 0.27 m/s during the base flow condition and 0.75 m/s at the wave peak), except near the bottom, due to the presence of the boundary layer. However, it should be noted that the proximity of a boundary may affect the ADV probe output, so that the streamwise velocity component can be underestimated [43]. The ADV and ADV Profiler have almost the same precision and accuracy. In fact, the mean value and standard deviation of the streamwise velocity do not significantly differ, regardless of the instrument used. In both cases, the relative errors are lower than 5% (error bars are not reported in Figure 7 for the sake of clarity). Specifically, the relative error is around 1–2% in the lower and intermediate part of the water column, while it increases in the upper part where the interaction between the wave and vegetation disturbs the signals.

**Figure 8:**
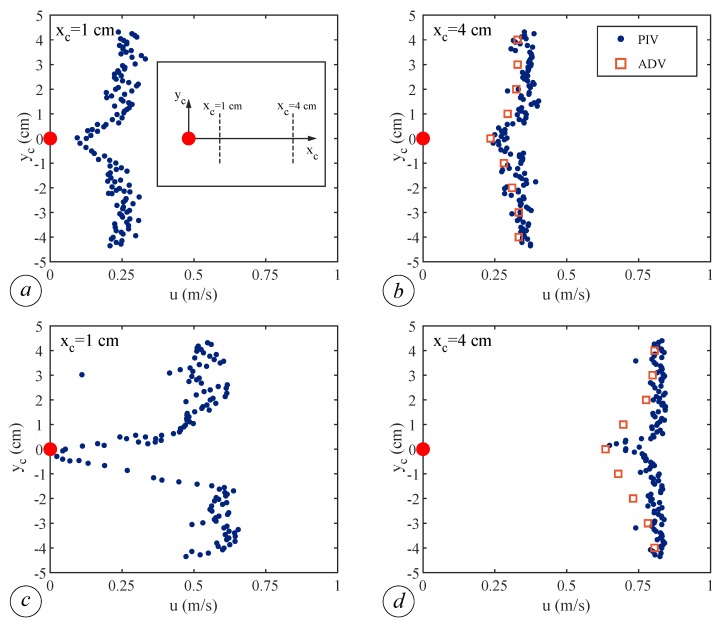
Transverse profiles of the longitudinal velocity component *u*, at x= 9.00 m and z= 2 cm, with vegetation density n=156.25 cylinder/m^2^. Base flow conditions at $x_{c}=1$ cm (**a**) and at $x_{c}=4$ cm (**b**). Wave peak conditions at $x_{c}=1$ cm (**c**) and at $x_{c}=4$ cm (**d**). The origin of the local reference system ($x_{c}$, $y_{c}$) coincides with the center of the cylinder, represented by a red dot (inset of Panel (**a**)).

The presence of vegetation affects the velocity distribution also in the transverse direction. In Figure 8, we report the transverse profiles of the longitudinal velocity *u* behind the cylinder located in the middle of the array with vegetation density of n=156.25 cylinder/m^2^, during base flow conditions and at the wave peak. The velocity profile reconstruction is carried out both using ADV and PIV measurements. The use of ADV is feasible only 4 cm apart from the rod, due to the minimum free volume required by the sensors to correctly measure the flow, whereas the PIV acquisition allows us to measure the flow velocity also very close to the cylinders and hence to overcome this drawback of the intrusive system. Figure 8b,d, where both ADV and PIV outputs are available in the same cross-sections, shows a very good agreement between the two velocity profiles, suggesting that the methodologies have likely the same accuracy. Such a result is also supported by the error analysis that gives a relative error of about 5% for ADV and of the same order of magnitude for PIV, thus making the two velocity measures equivalent. In both conditions, a relevant velocity reduction is detected downstream the cylinder, passing from 0.25 m/s to 0.10 m/s for the base flow (Figure 8a), and from 0.60 m/s to nearly 0 m/s at the wave peak (Figure 8c). The higher reduction of velocity observed between base flow and wave peak conditions can be ascribed to the larger wake area arising back at the cylinder.

**Figure 9:**
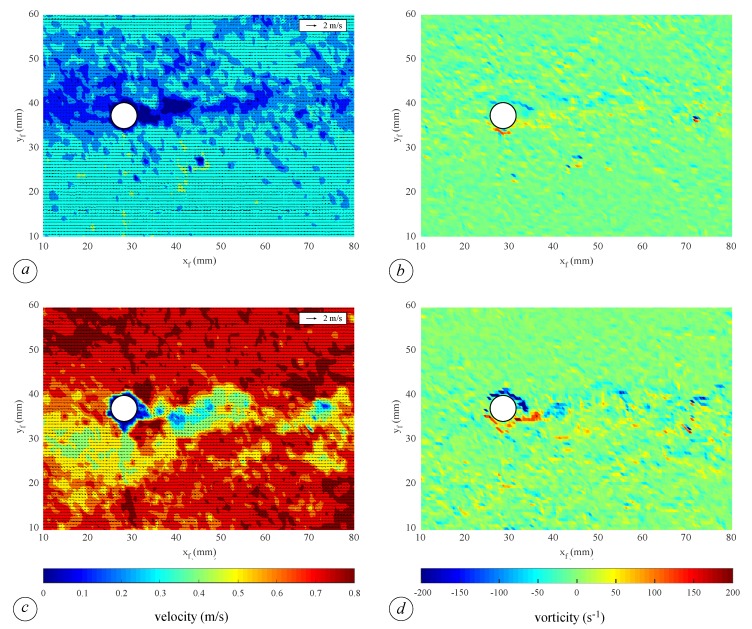
Example of PIV measurements at x= 9.00 m and z= 2 cm: velocity field during base flow condition (**a**) and at the wave peak (**c**); vorticity during base flow condition (**b**) and at the wave peak (**d**). The local coordinate system ($x_{f}$, $y_{f}$) refers to the frame. The flow is from left to right.

The flow velocity field obtained by means of the PIV technique confirms the latter result. Figure 9 shows the instantaneous horizontal velocity and vorticity field in the same domain analyzed in Figure 8. The velocity field for the base flow, as well as at the wave peak has heterogeneous low values downstream the cylinder (0÷0.1 m/s for the base flow and 0÷0.4 m/s at the passage of the wave), which transversally increases to an almost uniform higher value of 0.3 m/s and 0.75 m/s for the base flow and at the wave peak, respectively (see Figure 9a,c). Figure 9b,d shows the flow vorticity. The vertical component of the vorticity fluctuation is measured with the aim of relating it to the energy dissipation, hence with wave height attenuation. The vorticity map at the wave peak clearly shows two intense opposite vortexes downstream the cylinder, with vorticity ranging between −200 s^−1^ and 200 s^−1^ (Figure 9d). These values are almost four times greater than those estimated considering base flow conditions when the vorticity values range between −50 s^−1^ and 50 s^−1^ (Figure 9b). There are no substantial differences with the scenarios with higher vegetation density, save the mean value of the velocity.

## 4. Conclusions

This study presents an experimental setup properly designed for reproducing a solitary wave by an impulsive flow rate increase that is regulated by a software-controlled electro-valve. This approach overcomes the limits of the use of small waves and a cylinder array to represent the dissipation of flood-waves or tsunamis due to different types of vegetation in laboratory flumes.

In our experiments, water levels, point, and field velocity measurements have been carried out by means of ultrasonic probes, ADVs, an ADV Profiler, and a PIV system. The coupling of ADV and ultrasonic probes’ measurements allowed us to detect the velocity also during the significant rise of the water free surface, due to wave crest traveling. Moreover, the limits of ADV acquirements very close to the cylinders and to the walls were overcome by means of the non-intrusive nature of the PIV system. The results obtained using these different devices allowed us to reconstruct a detailed velocity field and, hence, the wake area arising back at the cylinder, quantifying the vorticity causing wave dissipation. Specifically, it was observed that the vegetation strongly affects the wave behavior, reducing the wave height proportionally with the vegetation density. During base flow conditions, the vertical velocity profile clearly showed a logarithmic profile in bare soil condition, whereas it turned into a rather uniform velocity distribution in the presence of vegetation, agreeing with many previous works (i.e., [40]). A wake area was observed downstream the cylinder rows, where a significant velocity reduction was noted both during base flow conditions and at the wave peak, even stronger in this second case. In fact, also the vorticity map field at the wave peak clearly showed the coexistence of two intense opposite eddies downstream the cylinder, with vorticity values almost four times greater than those estimated for the base flow condition.

The main aspects of the interaction between wave and vegetation can be fully obtained thanks to such a complete measurement system. However, the combined use of the different adopted sensors and techniques still has unexpressed potential in the cutting-edge investigation of this phenomenon and deserves further analysis.
